# Validity and reliability of deriving the autoregulatory plateau through projection pursuit regression from driven methods

**DOI:** 10.14814/phy2.15919

**Published:** 2024-01-23

**Authors:** Joel S. Burma, James K. Griffiths, Jonathan D. Smirl

**Affiliations:** ^1^ Cerebrovascular Concussion Lab, Faculty of Kinesiology University of Calgary Calgary Alberta Canada; ^2^ Sport Injury Prevention Research Centre, Faculty of Kinesiology University of Calgary Calgary Alberta Canada; ^3^ Human Performance Laboratory, Faculty of Kinesiology University of Calgary Calgary Alberta Canada; ^4^ Hotchkiss Brain Institute University of Calgary Calgary Alberta Canada; ^5^ Integrated Concussion Research Program University of Calgary Calgary Alberta Canada; ^6^ Alberta Children's Hospital Research Institute University of Calgary Calgary Alberta Canada; ^7^ Libin Cardiovascular Institute of Alberta University of Calgary Calgary Alberta Canada; ^8^ Faculty of Biomedical Engineering University of Calgary Calgary Alberta Canada

**Keywords:** cerebral autoregulation, oscillatory lower body negative pressure, projection pursuit regression, reliability, squat‐stand maneuvers, transcranial Doppler ultrasound

## Abstract

To compare the construct validity and between‐day reliability of projection pursuit regression (PPR) from oscillatory lower body negative pressure (OLBNP) and squat‐stand maneuvers (SSMs). Nineteen participants completed 5 min of OLBNP and SSMs at driven frequencies of 0.05 and 0.10 Hz across two visits. Autoregulatory plateaus were derived at both point‐estimates and across the cardiac cycle. Between‐day reliability was assessed with intraclass correlation coefficients (ICCs), Bland–Altman plots with 95% limits of agreement (LOA), coefficient of variation (CoV), and smallest real differences. Construct validity between OLBNP‐SSMs were quantified with Bland–Altman plots and Cohen's *d*. The expected autoregulatory curve with positive rising and negative falling slopes were present in only ~23% of the data. The between‐day reliability for the ICCs were poor‐to‐good with the CoV estimates ranging from ~50% to 70%. The 95% LOA were very wide with an average spread of ~450% for OLBNP and ~350% for SSMs. Plateaus were larger from SSMs compared to OLBNPs (moderate‐to‐large effect sizes). The cerebral pressure‐flow relationship is a complex regulatory process, and the “black‐box” nature of this system can make it challenging to quantify. The current data reveals PPR analysis does not always elicit a clear‐cut central plateau with distinctive rising/falling slopes.

## 1 INTRODUCTION

Cerebral autoregulation (CA) refers to the ability of the cerebrovasculature to adapt to systemic changes in blood pressure, ensuring cerebral perfusion remains intact (Brassard et al., 2021; Lassen, 1959). In the 1950s, this concept was proposed with cerebral perfusion remaining constant over a large range of blood pressure values (i.e., 60–150 mmHg), known as Lassen's curve (Lassen, 1959). These early investigations relied on the Kety‐Schmidt technique, where it takes upward of 20‐min to obtain a single assessment of cerebral blood flow (Kety & Schmidt, 1945). However, the development of transcranial Doppler ultrasound (TCD) allowed for beat‐to‐beat assessments between cerebral blood velocity (CBv) and blood pressure (Aaslid et al., 1989). This has unveiled the wide range of autoregulation is not accurate, rather a narrow autoregulation plateau of ~5–10 mmHg exists with substantial step gradients on either side (Brassard et al., 2021).

This updated CA curve, with a very narrow autoregulatory region, was first demonstrated by the work of Tan, Hamner, and colleagues through the use of projection pursuit regression (PPR) (Hamner & Tan, 2014; Tan, 2012; Tan et al., 2013; Taylor et al., 2014). This technique is a non‐parametric and multiple‐regressive atheoretical method that is capable of determining the nonlinear patterns within the cerebral pressure‐flow relationship (Hamner & Tan, 2014; Tan, 2012; Tan et al., 2013; Taylor et al., 2014). These studies using PPR have primarily used oscillatory lower body negative pressure (OLBNP) to induce blood pressure oscillations (~10–20 mmHg) to augment the coherency between blood pressure and CBv (Hamner & Tan, 2014; Tan, 2012; Tan et al., 2013; Taylor et al., 2014). However, numerous other driven techniques have been used to measure CA, including squat‐stand maneuvers (SSMs), which produce pressure oscillations upward of ~30–50 mmHg (Barnes et al., 2018; Batterham et al., 2020; Burma, Copeland, Macaulay, Khatra, Wright, & Smirl, 2020; Newel et al., 2022; Panerai et al., 2021; Smirl et al., 2015). Moreover, previous investigations using SSMs have established cardiac cycle differences are present with greater regulation occurring in systole compared to diastole (Burma, Copeland, Macaulay, Khatra, & Smirl, 2020; Burma, Copeland, Macaulay, Khatra, Wright, & Smirl, 2020; Newel et al., 2022; Smirl et al., 2018; Wright et al., 2018). However, these studies were conducted using transfer functional analysis (TFA) independently (Burma, Copeland, Macaulay, Khatra, & Smirl, 2020; Burma, Copeland, Macaulay, Khatra, Wright, & Smirl, 2020; Newel et al., 2022; Smirl et al., 2018; Wright et al., 2018).

Therefore, the purpose of the current investigation was to expand upon the previous PPR research by exploring the construct validity of deriving these estimates from SSMs against OLBNP (i.e., the current “gold‐standard” for PPR) (Hartmann et al., 2023). The initial PPR work only examined the mean cerebral pressure‐flow relationship (Tan, 2012), and thus the current paper sought to expand these investigations by employing a similar analysis in the diastolic and systolic components of the cardiac cycle. Moreover, the reliability of this technique has only been quantified with a very small subsample of only five participants (Tan, 2012). Thus, the between‐day reliability estimates from both OLBNP and SSMs across the cardiac cycle will be explored to understand the validity of comparisons within longitudinal/repeated measure study designs (Hartmann et al., 2023). This will further highlight the potential physiological utility of this atheoretical technique to identify abnormal CA in clinical populations. Based on the previous PPR findings, it was hypothesized the autoregulatory plateau will show greater reliability at 0.05 Hz compared to 0.10 Hz, due to greater counter‐regulation occurring at lower frequencies (Tan, 2012). Moreover, it was hypothesized metrics derived from SSMs would display greater reliability compared to those from OLBNP, resulting from the larger blood pressure oscillations SSMs are known to produce (Smirl et al., 2015). Finally, it was hypothesized the construct validity to be similar between both driven methods when deriving the autoregulatory plateau from PPR approaches.

## 2 METHODS

### 2.1 Ethical approval

Data used in the current investigation were from a previously published investigation that examined dynamic CA (dCA) within the frequency domain through linear TFA (Smirl et al., 2015). This experimentation was given ethical approval from the Clinical Ethical Committees of the Universities of British Columbia and aside from registration within a database, followed the principles within the Declaration of Helsinki. Prior to data collection, all participants provided written informed consent.

### 2.2 Participants

Data were collected from 19 participants (2 females and 17 males), including 10 younger participants (0 females and 10 males; age: 24.8 ± 2.7 years; body mass index: 23.6 ± 2.4 kg/m^2^) and 9 older participants (2 females and 7 males; 66.4 ± 3.7 years; body mass index of 25.6 ± 2.7 kg/m^2^). Older participants were screened for the absence of artery stenosis and occlusion by a trained physician. All participants had no history of any medical conditions/complications and refrained from caffeine, exercise, alcohol, and smoking for a minimum of 12‐h prior to data collection (Burma, Copeland, Macaulay, Khatra, Wright, & Smirl, 2020; Kennedy et al., 2022).

### 2.3 Instrumentation

Bilateral middle cerebral artery velocity (MCAv) was quantified through TCD via two 2‐MHz Doppler ultrasound probes (Spencer Technologies, Seattle, WA). Trained sonographers ensured the middle cerebral artery was insonated based on established velocities, waveforms, and signal depths (Willie et al., 2014). Participants were also attached to a Finometer to quantify blood pressure on a beat‐to‐beat basis (Finometer; Finapres Medical Systems, Amsterdam, The Netherlands). This device uses finger photoplethysmography and corrects for the height of the heart with a height correct unit, which has been shown to have high levels of reliability with intra‐arterial blood pressure (Omboni et al., 1993; Sammons et al., 2007). Partial pressure values of carbon dioxide and oxygen were quantified on a breath‐by‐breath basis to ensure all participants remained within eucapnia during the autoregulatory assessments (ML206; ADInstruments, Colorado Springs, CO). Three lead electrocardiography captured R‐R intervals using lead II methodology. Data were sampled at 1000 Hz with LabChart version 7.1 (ADInstruments, Colorado Springs, CO), which allowed for offline analysis.

### 2.4 Experimental protocols

Data were collected over two testing sessions within a range of 48‐ to 144‐h and were performed at the same time of day in order to minimize any potential effects of diurnal variation (Burma, Copeland, Macaulay, Khatra, & Smirl, 2020; Conroy et al., 2005; Labrecque et al., 2022). Participants completed SSMs at both 0.05 and 0.10 Hz for 5 min in a randomized order (Burma et al., 2021). During these maneuvers, participants squatted until a ~90° angle was formed between the upper and lower legs (Claassen et al., 2009). This was followed by OLBNP assessments at the same frequencies of interest, which the previous PPR studies have noted represent myogenic (Hamner et al., 2010; Hamner & Tan, 2014; Tan et al., 2013) (i.e., 0.05 Hz) and sympathetic (Julien, 2006) (0.10 Hz) influences on the cerebrovasculature. The OLBNP oscillations occurred between 0 and −50 Torr with transitions happening within 2–3 s (Formes et al., 2010).

### 2.5 Data processing and projection pursuit regression

Data were processed using RStudio (Version 2023.06.0) in accordance with the previous PPR studies (Hamner & Tan, 2014; Tan, 2012; Tan et al., 2013; Taylor et al., 2014). Specifically, all PPR analysis was completed based on the detailed PPR methods paper (Taylor et al., 2014). First, data were downsampled to 5 Hz and low‐pass filtered with a cutoff of 0.4 Hz (Taylor et al., 2014). Following data were band‐passed filtered, around the two oscillation frequencies of interest (i.e., 0.05 and 0.10 Hz) in a ± 0.005 Hz band (Taylor et al., 2014). The relationship between blood pressure and MCAv was then characterized using PPR. Briefly, PPR is a nonparametric, multiple‐regression method that iteratively sums a linear combination of ridge terms (Friedman & Stuetzle, 1981). A single ridge function was used as utilizing more than one can reduce the ability to derive physiological interpretations from the data due to potential interactions between ridge functions (Taylor et al., 2014). Consistent with the methods laid out in (Taylor et al., 2014), piecewise linear parameterization was achieved via free‐knot splines to separate ridge functions into three linear regions (Taylor et al., 2014). This demarcated the regions where the pressure‐flow relationship differed from zero (i.e., rising and falling slopes) and the region where the relationship would be linear (i.e., autoregulatory plateau). The distance between the two knots separating these regions was interpreted as the autoregulatory plateau. The main outcome of interest was the autoregulatory plateau based on the findings from Saleem and colleagues (Saleem et al., 2015) who noted several subjects displayed an autoregulatory region; however, only 28% displayed an expected autoregulatory plateau with negative falling slopes and positive rising slopes.

Linear TFA estimates were computed according to published recommendations (Claassen et al., 2016; Panerai et al., 2023). In brief, beat‐to‐beat blood pressure and CBv waveforms were spline interpolated using a Welch smoothing method and resampled at 4 Hz. The 5‐min of data were detrended and passed through five Hanning windows with 50% overlap (i.e., 100‐s per window). Cross‐spectrums for each cardiac cycle phase were calculated and divided by the respective autospectrum. Cerebral pressure‐flow TFA estimates were then computed at 0.05 and 0.10 Hz, the driven point‐estimates of interest. TFA outcome metrics included the power spectral densities, coherence, phase, gain, and normalized gain (Burma, Copeland, Macaulay, Khatra, & Smirl, 2020; Smirl et al., 2015; Zhang et al., 1998). The a priori critical coherence value was determined using an alpha of 0.01, resulting in a coherence threshold of 0.46 (Panerai et al., 2023). No datasets contained phase wraparound.

### 2.6 Statistical analysis

All analyses were completed in RStudio (Version 2023.06.0). The between‐day reliability and construct validity were completed within younger and older cohorts and collapsed across both (i.e., pooled estimates). To assess the between‐day reliability of the autoregulatory plateau estimates, Wilcoxon signed‐ranked tests were first used to identify the presence of systemic error at the group level that would make test–retest reliability analyses unsuitable (Hartmann et al., 2023). Wilcoxon tests were used due to the small sample size. Relative reliability was calculated with intraclass correlation coefficients (ICC) and the associated 95% confidence intervals (95% CI) and coefficient of variation (CoV) estimates and 95% CI (Hartmann et al., 2023). Absolute reliability was calculated through Bland–Altman Plots with 95% limits of agreement (LOA) and the smallest real difference (Hartmann et al., 2023). The ICC metrics were computed using two‐way random effects, absolute agreement, and single rater/measurement [i.e., ICC (Brassard et al., 2021; Lassen, 1959)] with a priori thresholds of 0.00–0.50 (poor), 0.50–0.75 (moderate), 0.75–0.90 (good), and 0.90–1.00 (excellent) (Koo & Li, 2016). Some negative ICC values were encountered, which were converted to zeros for interpretation. Negative values can occur when variability within subjects exceeds the variability between subjects (Liljequist et al., 2019). The CoV was computed as the quotient between the standard deviation and the mean values of the autoregulatory plateau. The associated 95% CI for the CoV estimates were calculated in accordance with published recommendations (Hartmann et al., 2023). CoV thresholds consisted of 0%–5% (excellent), 5%–10% (good), 10%–20% (moderate), and >20% (poor) (Burma et al., 2022). Giavarina (2015) has extensively shown the inability of Bland–Altman analysis to be completed on non‐normally distributed data, due to artificial bias. Therefore, differences (i.e., y‐axis) were expressed as ratios (percent) to control for the skewed data (Giavarina, 2015). Finally, the smallest real difference (SRD) was calculated for the between‐day reliability analysis using the following formula, where SDw is the standard deviation within the participants. The 2.101 was the critical *t*‐value determined based on an alpha of 0.05 and a degree of freedom of 18.

$$\mathrm{SRD}=2.101*\sqrt{2{\left(\mathrm{SDw}\right)}^{2}}$$

To understand the construct validity of obtaining the autoregulatory plateau via SSMs, these values were compared to values obtained from the current “gold‐standard” in PPR analyses (i.e., OLBNP) using Bland–Altman plots with 95% LOA and COV metrics. These comparisons followed the same principles described above. In addition, the magnitude difference of the autoregulatory plateau was determined using Cohen's *d* effect sizes with thresholds of negligible (<0.20), small (0.20–0.50), moderate (0.50–0.80), and large (>0.80) (Lakens, 2013). Effect sizes were utilized as these have been proposed to provide clinical relevance in physiological literature to a greater extent than a binary *p*‐value (Amrhein et al., 2019; Halsey, 2019; Panagiotakos, 2008). Finally, to understand if the variability could be explained by a poor dataset, rather than an analytical approach, the association between the autoregulatory range and TFA estimates were computed using a coefficient of determination (*r* ^2^) via simple linear regression. Alpha was set a priori at 0.05.

## 3 RESULTS

**FIGURE 1:**
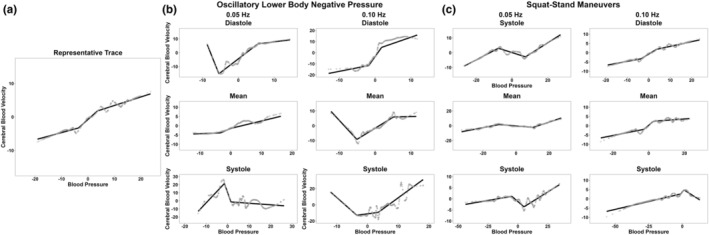
A representation trace of the PPR Curve (a), the PPR data produced from OLBNP (b), and the PPR data produced from SSMs (c) from one individual. These were derived at both 0.05 and 0.10 Hz, as well as across the three phases of the cardiac cycle (diastole, mean, and systole).

The average autoregulatory plateau ranges are displayed in Table 1, stratified for age. Figure 1 displays representative data of the PPR curve (Figure 1a), and the actual results derived from OLBNP (Figure 1b) and SSMs (Figure 1c) from one individual. Collectively, a total of 456 PPR plots were computed, where 103 (22.6%) produced a figure resembling an autoregulatory plateau with a rising and falling slope (Figure 1a). SSMs produced an expected autoregulatory curve 28.5% of the time, while OLBNP produced a curve in 16.7% of the data. A curve was produced in 27.2% and 18.0% of the data at 0.05 and 0.10 Hz, respectively. Finally, diastole produced a curve 30.9%, mean 19.1%, and systole 17.8% of the time.

**TABLE 1:** The average autoregulatory plateau range (measured in millimeters of mercury) derived from projection pursuit regression analysis within 19 individuals (2 females and 17 males) during oscillatory lower‐body negative pressure (OLBNP) and squat‐stand maneuvers (SSMs).

Frequency	Cycle	Age	OLBNP	SSM	Cohen's *d* (95% CI)	Magnitude
0.05 Hz	Diastole	All	3.52 ± 3.74	10.74 ± 10.79	−0.89 (−1.29, −0.55)	Large
		Older	4.37 ± 4.18	9.53 ± 11.66	−0.59 (−1.11, 0.03)	Moderate
		Younger	2.75 ± 3.22	11.83 ± 10.12	−1.21 (−1.89, −0.78)	Large
	Mean	All	4.87 ± 4.74	11.03 ± 11.42	−0.70 (−1.09, −0.35)	Moderate
		Older	6.68 ± 5.64	11.62 ± 13.81	−0.47 (−0.96, 0.23)	Small
		Younger	3.25 ± 3.07	10.51 ± 9.10	−1.07 (−1.61, −0.61)	Large
	Systole	All	7.68 ± 7.53	17.58 ± 15.38	−0.82 (−1.27, −0.45)	Large
		Older	10.03 ± 8.28	17.67 ± 14.60	−0.64 (−1.33, 0.03)	Moderate
		Younger	5.57 ± 6.25	17.50 ± 16.43	−0.96 (−1.54, −0.55)	Large
0.10 Hz	Diastole	All	3.19 ± 3.34	13.00 ± 7.33	−1.72 (−2.32, −1.29)	Large
		Older	2.87 ± 2.56	9.79 ± 5.31	−1.66 (−2.69, −1.10)	Large
		Younger	3.47 ± 3.95	15.89 ± 7.80	−2.01 (−3.08, −1.35)	Large
	Mean	All	4.71 ± 5.24	10.69 ± 9.51	−0.78 (−1.26, −0.34)	Moderate
		Older	5.54 ± 6.46	6.39 ± 5.37	−0.14 (−1.11, 0.49)	Negligible
		Younger	3.96 ± 3.87	14.55 ± 10.82	−1.30 (−2.05, −0.76)	Large
	Systole	All	6.34 ± 6.87	10.82 ± 11.08	−0.49 (−0.92, −0.04)	Small
		Older	6.61 ± 6.24	9.51 ± 10.06	−0.35 (−1.11, 0.36)	Small
		Younger	6.10 ± 7.54	12.00 ± 12.05	−0.59 (−1.22, 0.02)	Moderate

*Note*: Data are displayed as mean ± standard deviation. Cohen's *d* effect size thresholds of <0.20 (negligible), 0.20–0.50 (small), 0.50–0.80 (moderate), >0.80 (large) were used. Data were stratified into older (*n* = 9; 2 females and 7 males) and younger (*n* = 10; 0 females and 10 males) participants.

**FIGURE 2:**
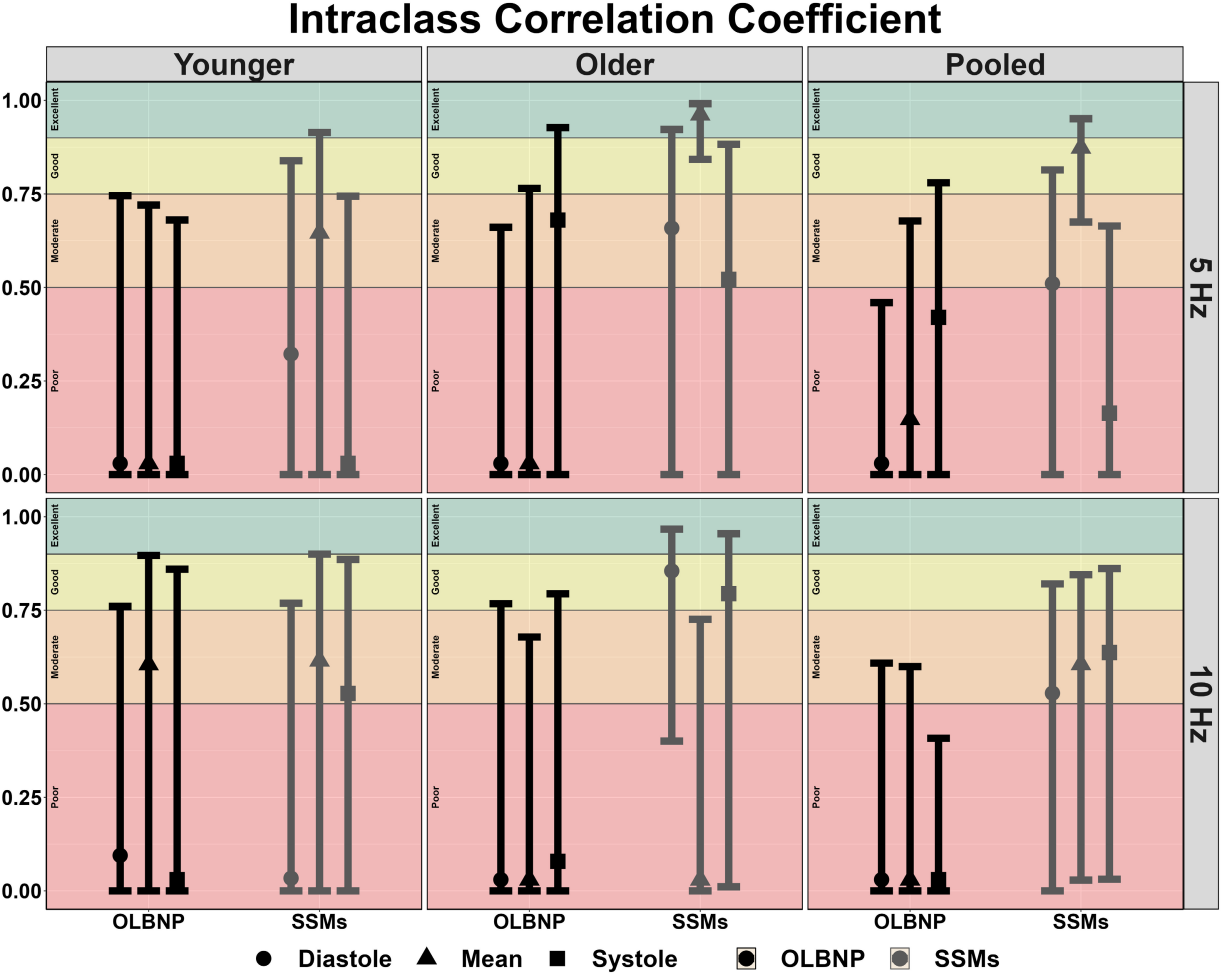
Between‐day intraclass correlation coefficients (ICC) for the autoregulatory plateau derived through projection pursuit analysis via oscillatory lower body negative pressure (OLBNP; black) and squat‐stand maneuvers (SSMs; gray) at 0.05 and 0.10 Hz. These were calculated in 19 participants (2 female, 17 male) across the three phases of the cardiac cycle (i.e., diastole, mean, and systole), and stratified into older (*n* = 9; 2 females and 7 males), younger (*n* = 10; 0 females and 10 males), and combined (i.e., pooled) participants. The ICC metrics were computed using two‐way random effects, absolute agreement, and single rater/measurement (i.e., ICC (Brassard et al., 2021; Lassen, 1959)) with a priori thresholds of 0.00–0.50 (poor), 0.50–0.75 (moderate), 0.75–0.90 (good), and 0.90–1.00 (excellent) (Koo & Li, 2016). Negative ICC values were encountered, which were converted to zeros for interpretation. Negative values can occur when variability within subjects exceeds the variability between subjects (Liljequist et al., 2019). Data are presented as mean with 95% confidence intervals; however, individual data points are not presented as this is not possible to obtain with ICC calculations.

**FIGURE 3:**
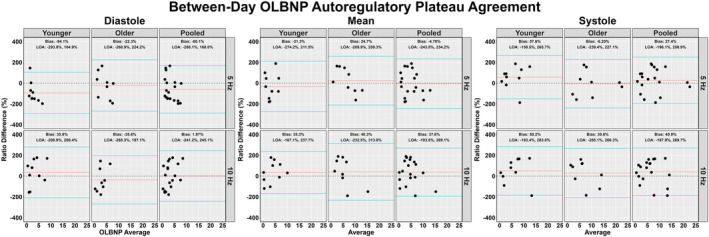
Bland–Altman plot with 95% limits of agreement depicting the between‐day reliability of deriving the autoregulatory plateau through projection pursuit analysis via oscillatory lower body negative pressure (OLBNP) at 0.05 and 0.10 Hz. These were calculated in 19 participants (2 female, 17 male) across the three phases of the cardiac cycle (i.e., diastole, mean, and systole), and stratified into older (*n* = 9; 2 females and 7 males), younger (*n* = 10; 0 females and 10 males), and combined (i.e., pooled) participants. Differences were transformed into ratios due to non‐normally distributed data.

**FIGURE 4:**
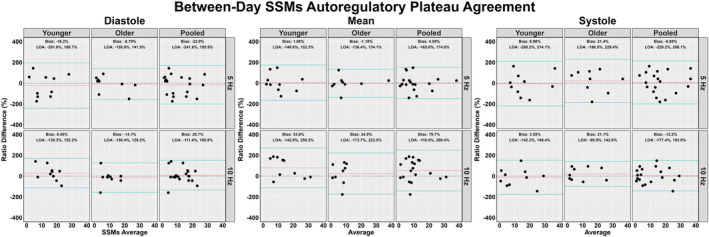
Bland–Altman plot with 95% limits of agreement depicting the between‐day reliability of deriving the autoregulatory plateau through projection pursuit analysis via squat‐stand maneuvers (SSMs) at 0.05 and 0.10 Hz. These were calculated in 19 participants (2 female, 17 male) across the three phases of the cardiac cycle (i.e., diastole, mean, and systole), and stratified into older (*n* = 9; 2 females and 7 males), younger (*n* = 10; 0 females and 10 males), and combined (i.e., pooled) participants. Differences were transformed into ratios due to non‐normally distributed data.

**FIGURE 5:**
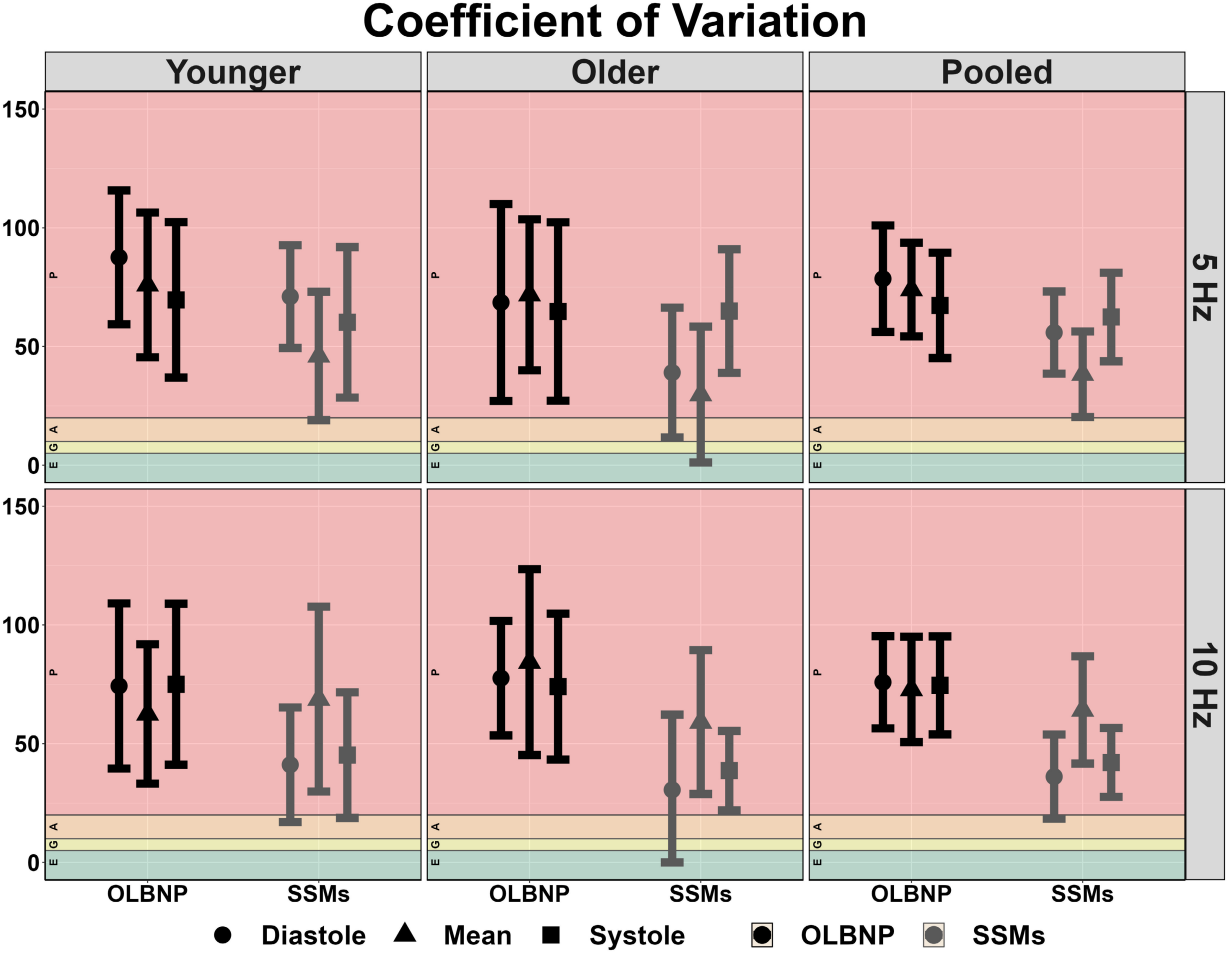
Between‐day coefficient of variation for the autoregulatory plateau derived through projection pursuit analysis via oscillatory lower body negative pressure (OLBNP; black) and squat‐stand maneuvers (SSMs; gray) at 0.05 and 0.10 Hz. These were calculated in 19 participants (2 female, 17 male) across the three phases of the cardiac cycle (i.e., diastole, mean, and systole), and stratified into older (*n* = 9; 2 females and 7 males), younger (*n* = 10; 0 females and 10 males), and combined (i.e., pooled) participants. The CoV was computed as the quotient between the standard deviation and the mean values of the autoregulatory plateau with thresholds of 0%–5% (excellent), 5%–10% (good), 10%–20% (moderate), and >20% (poor) (Burma et al., 2022). Data are presented as mean with 95% confidence intervals with individual data points presented.

The between‐day reliability of both techniques was poor for all cardiac cycle components and across both frequencies of interest (Figures 2, 3, 4, 5). The average ICC estimates and 95% CI collapsed between driven methods were 0.33 (95% CI: 0.06, 0.71); however, these were slightly higher for SSMs (0.55; 95% CI: 0.12, 0.83) compared to OLBNP (0.11; 95% CI: 0.00, 0.59) (Figure 2). For the between‐day reliability, the mean bias from the Bland–Altman plots were: −29.1% for OLBNP diastole, −3.85% for SSMs diastole, 16.4% for OLBNP mean, −27.8% for SSMs mean, 34.1% for OLBNP systole, and 5.26% for SSMs systole (Figures 3 and 4). However, the 95% LOA were very wide with a total spread of: 471% for OLBNP diastole, 329% for SSMs diastole, 470% for OLBNP mean, 347% for SSMs mean, 452% for OLBNP systole, and 353% for SSMs systole (Figures 3 and 4). The averaged pooled CoV estimates collapsed between driven methods were 61.8% (95% CI: 49.8, 73.8%); however, these were slightly lower for SSMs (52.0%; 95% CI: 40.8, 63.3%) compared to OLBNP (71.6%; 95% CI: 58.9, 84.4%) (Figure 5). The CoV 95% CI fell within the poor range for all OLBNP measures and acceptable‐to‐poor for SSMs (Figure 5). The SRD values are displayed in Table 2, with the majority of these being larger than the autoregulatory values produced in Table 1.

**TABLE 2:** The smallest real difference calculated average autoregulatory plateau range (measured in millimeters of mercury) derived from projection pursuit regression analysis within 19 individuals (2 females and 17 males) during oscillatory lower‐body negative pressure (OLBNP) and squat‐stand maneuvers (SSMs).

Method	Frequency	Cycle	Older	Younger	Pooled
OLBNP	5 Hz	Diastole	10.9	8.1	9.3
		Mean	9.8	5.9	8.5
		Systole	11.2	15.1	13.0
	10 Hz	Diastole	4.7	9.4	7.4
		Mean	15.3	6.2	11.9
		Systole	12.2	18.9	15.7
SSMs	5 Hz	Diastole	23.2	14.5	19.4
		Mean	9.1	13.3	11.9
		Systole	22.5	37.1	30.2
	10 Hz	Diastole	6.0	14.2	12.6
		Mean	11.0	15.2	13.6
		Systole	13.2	24.6	19.6

*Note*: Data were stratified into older (*n* = 9; 2 females and 7 males) and younger (*n* = 10; 0 females and 10 males) participants.

**FIGURE 6:**
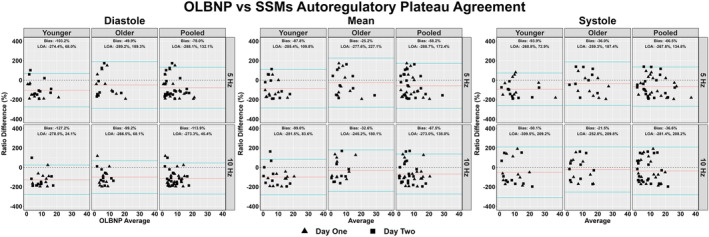
Bland–Altman plot with 95% limits of agreement displaying construct validity of deriving the autoregulatory plateau through projection pursuit analysis via oscillatory lower body negative pressure (OLBNP) and squat‐stand maneuvers (SSMs) at 0.05 and 0.10 Hz. These were calculated in 19 participants (2 female, 17 male) across the three phases of the cardiac cycle (i.e., diastole, mean, and systole), and stratified into older (*n* = 9; 2 females and 7 males), younger (*n* = 10; 0 females and 10 males), and combined (i.e., pooled) participants. Differences were transformed into ratios due to non‐normally distributed data.

**FIGURE 7:**
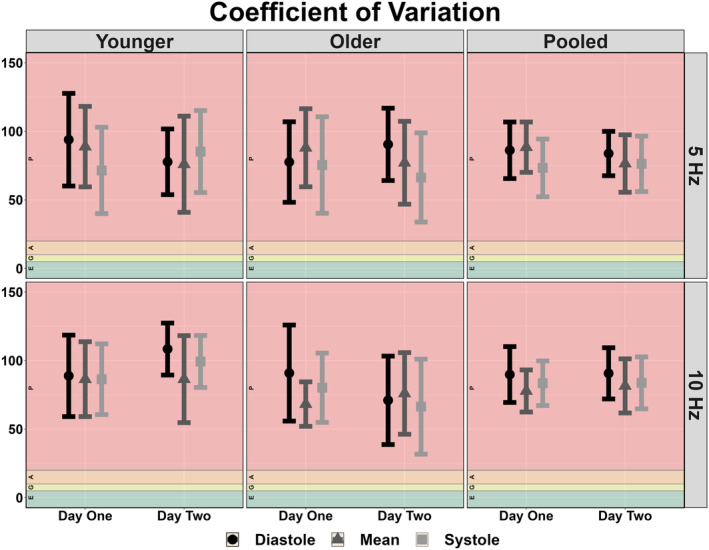
Coefficient of variation displaying the construct validity of deriving the autoregulatory plateau through projection pursuit analysis comparing oscillatory lower body negative pressure and squat‐stand maneuvers at 0.05 and 0.10 Hz. These were calculated in 19 participants (2 female, 17 male) across the three phases of the cardiac cycle (i.e., diastole, mean, and systole), and stratified into older (*n* = 9; 2 females and 7 males), younger (*n* = 10; 0 females and 10 males), and combined (i.e., pooled) participants. The CoV was computed as the quotient between the standard deviation and the mean values of the autoregulatory plateau with thresholds of 0%–5% (excellent), 5%–10% (good), 10%–20% (moderate), and >20% (poor) (Burma et al., 2022). Data are presented as mean with 95% confidence intervals with individual data points presented.

Due to the poor between‐day reliability, data were not collapsed within participants between days and thus the comparison between OLBNP and SSMs were completed based on values obtained from both days. All plateaus were larger when produced from the SSMs compared to OLBNPs, with several of these eliciting moderate‐to‐large effect sizes (78%) (Table 1). Values collapsed across frequency and day produced autoregulatory plateau values of diastole OLBNP: 3.35 ± 3.52 mmHg, diastole SSMs: 11.9 ± 9.23 mmHg; mean OLBNP: 4.79 ± 4.96 mmHg, mean SSMs: 10.9 ± 10.4 mmHg; and systole OLBNP: 7.01 ± 7.19 mmHg, systole SSMs: 14.2 ± 13.7 mmHg. Values collapsed across cardiac cycle component and day were 0.05 Hz OLBNP: 5.36 ± 5.79 mmHg, 0.05 Hz SSMs: 13.1 ± 13.0 mmHg; and 0.05 Hz OLBNP: 4.75 ± 5.45 mmHg, 0.10 Hz SSMs: 11.5 ± 9.41 mmHg. For the construct validity, a negative bias was present for all comparisons with SSMs producing a larger autoregulatory plateau: −78% for 0.05 Hz diastole, −114% for 0.10 Hz diastole, −58% for 0.05 Hz mean, −68% for 0.10 Hz mean, −67% for 0.05 Hz systole, −37% for 0.10 Hz systole (Figure 6). These additionally displayed a wide 95% LOA with a total spread of: 420% for OLBNP diastole, 461% for SSMs diastole, 403% for OLBNP mean, 319% for SSMs mean, 411% for OLBNP systole, and 490% for SSMs systole (Figures 6). Finally, the coefficient of variation estimate and 95% CI were poor for all comparisons between OLBNP and SSMs (Figure 7).

Table 3 displays the between‐day variability of the autoregulatory plateau derived via PPR and its association with the between‐day variability of the TFA outcome metrics. The median explained variance between analytical approaches was 4% (interquartile range: 1%–11%) (Table 3), highlighting the variability was not a function of a poor dataset but rather than analytical approach.

**TABLE 3:** Adjusted *r* ^2^ values comparing the coefficient of variation between the autoregulatory plateau and transfer functional analysis variables in 19 individuals (2 females and 17 males) during oscillatory lower‐body negative pressure (OLBNP) and squat‐stand maneuvers (SSMs).

Method	Frequency	Cycle	BP PSD	MCA_PSD	Coherence	Phase	Gain	nGain
OLBNP	0.05 Hz	Diastole	0.46	0.16	0.34	0.14	0.05	0.07
		Mean	0.01	0.46	0.11	0.00	0.01	0.00
		Systole	0.10	0.04	0.02	0.05	0.00	0.00
	0.10 Hz	Diastole	0.12	0.05	0.00	0.01	0.01	0.15
		Mean	0.01	0.50	0.00	0.16	0.15	0.07
		Systole	0.00	0.15	0.01	0.00	0.09	0.02
SSM	0.05 Hz	Diastole	0.02	0.02	0.23	0.13	0.02	0.09
		Mean	0.00	0.00	0.01	0.00	0.02	0.00
		Systole	0.10	0.16	0.00	0.09	0.21	0.03
	0.10 Hz	Diastole	0.07	0.21	0.00	0.01	0.00	0.05
		Mean	0.04	0.03	0.56	0.02	0.07	0.18
		Systole	0.10	0.04	0.00	0.08	0.05	0.00

## 4 DISCUSSION

The current investigation aimed to better understand the utility of PPR by deriving the autoregulatory plateau across the cardiac cycle (i.e., diastole, mean, and systole), while assessing the between‐day reliability when using different driven approaches (i.e., OLBNP and SSMs), and determining the construct validity between SSMs and the current “gold‐standard” OLBNP. The main findings were: (1) the autoregulatory plateaus were the greatest in systole and smallest in diastole; (2) an expected autoregulatory curve with a positive rising slope and a negative falling slope occurred in only ~23% of the traces; (3) the between‐day reliability for both methods used to produce an autoregulatory plateau was poor; (4) a systematic bias was noted between methods with SSMs producing a greater plateau compared to OLBNP. These results were in agreement with the findings by Saleem and colleagues (Saleem et al., 2015) who noted only 28% of their dataset displayed an autoregulatory curve with a plateau, rising and falling slopes. Hence, interpretations derived from PPR should be taken with a degree of caution due to the inherently poor levels of reproducibility.

A previous report by Saleem et al. (2015), sought to characterize the relationship between blood pressure and CBv using OLBNP at 0.03, 0.05, and 0.07 Hz through both PPR and locally weighted scatterplot smoother plots. At all three frequencies of interest, the authors found a heterogeneous pattern with only ~28% of the datasets producing a dCA autoregulatory plateau with rising and falling slopes. Similarly, this investigation found that ~23% displayed the expected autoregulatory plateau, which was slightly more apparent at 0.05 Hz (27.2%) compared to 0.10 Hz (18.0%). This means ~70%–80% of participants did not display the expected CA curve produced through PPR analyses, within two independent investigations. The inconsistency of eliciting a robust autoregulatory plateau likely underpins the poor between‐day reliability seen for all comparisons (Figures 2, 3, 4, 5). A common issue with dCA metrics in general is the poor reproducibility elicited (Sanders et al., 2018; Sanders et al., 2019) For example, several previous reports have demonstrated spontaneous TFA metrics to produce poor‐to‐fair levels of reproducibility (Burma, Copeland, Macaulay, Khatra, & Smirl, 2020; Smirl et al., 2015), in addition to other methodological approaches such as autoregulatory index and correlation coefficient analyses (Sanders et al., 2018). Nevertheless, the same dataset from the current investigation was used previously for TFA estimates, where the CoV generally ranged from ~20% to 40% for spontaneous and OLBNP methods and was ~10%–20% for SSMs (Smirl et al., 2015). Conversely, the between‐day CoV in the current investigation generally ranged between 50% and 70% (Figure 5). The coefficient of determination analysis in Table 3 sought to explore if the poor reliability in the autoregulatory plateau corresponded to the TFA estimates that also displayed greater variability. However, minimal association was found, highlighting this was likely not due to the nature of the data collected but rather the analytical approach (Table 3). The larger CoV also translated to large SRD values that exceeded the absolute autoregulatory plateau values (Table 2). In a clinical context, a large SRD value indicates that a substantial change in a patient's condition or response to treatment is required to distinguish it from random variability or measurement error. This suggests that relatively small fluctuations in PPR may not be clinically significant or meaningful. A potential explanation for the poorer reproducibility may be due to the poor test–retest reliability of using non‐invasive blood pressure assessments (i.e., Finapres) (Olsen et al., 2022). Nevertheless, this is unlikely as the same blood pressure data produced acceptable levels of between‐day reliability between older and younger participants in TFA metrics derived from SSMs (Smirl et al., 2015). Further, other investigations have demonstrated the Finapres to elicit high levels of within‐ and between‐day reliability (Burma et al., 2021; Burma, Copeland, Macaulay, Khatra, & Smirl, 2020; Burma, Copeland, Macaulay, Khatra, Wright, & Smirl, 2020). Of interest, despite to poor reliability, a greater plateau was consistently noted when derived via SSMs compared to OLBNP. This may be attributable to the differing fluctuations in blood pressure, where SSMs are known to elicit ~30–50 mmHg oscillations, while OLBNP produces ~10–20 mmHg (Smirl et al., 2015). However, more research into this is warranted.

This study followed guidelines for performing PPR as put forth by Taylor et al. (2014), with respect to CA investigations. Using this method, previous papers have suggested the cerebrovasculature is only capable of dampening oscillations slower than 0.07 Hz, but those faster than this pass through the cerebrovasculature relatively unimpeded (Tan et al., 2013). The previous PPR works by Tan and colleagues have suggested this is due to the absence of an autoregulatory plateau between the rising and falling slopes above 0.07 Hz (Hamner & Tan, 2014; Tan, 2012; Tan et al., 2013; Taylor et al., 2014). However, the extensive work in the CA field performed via TFA analysis in the frequency domain have suggested the upper‐frequency limit is much higher and expected to occur closer to ~0.20 Hz. This higher frequency limit for CA regulation from the CARNet community of 0.20 Hz is the threshold where the input (systemic blood pressure) and output (CBv) are associated with a gain value of ~1.0 cm/s/mmHg and a phase of 0.0 radians (or degrees) (Claassen et al., 2016; Panerai et al., 2023). The former means the amplitude change of blood pressure being passed through the cerebrovasculature is similar between the systemic (body) and central (the brain) regions, while the latter means there is virtually no timing offset between the brain and body (Zhang et al., 1998). This was originally demonstrated by the seminal work by Zhang et al (1998), who were the first to assess the cerebral pressure‐flow relationship using TFA. These authors noted that while the gain was approximately 1.0 at a value of 0.10 Hz, the phase gradually decreased until a threshold of ~0.20 Hz (Zhang et al., 1998). Nonetheless, the cerebral pressure‐flow relationship is a complex process with numerous intricacies and thus stating a singular value as an upper‐frequency limit for all individuals is likely an oversimplification. For example, Hamner et al. (2019) utilized OLBNP demonstrating near complete counter‐regulation occurred below a frequency of 0.03 Hz. Within the low‐frequency band ranging 0.03–0.10 Hz, the counter‐regulation was not nearly as efficient; however, the dampening of the blood pressure oscillations was present (gain ~0.5 cm/s/mmHg). This study nonetheless insonated the middle cerebral artery independently, while only using gain values, where previous work denoted the upper‐frequency limit of ~0.20 Hz is attributable to the timing buffer disappearing (i.e., phase) (Zhang et al., 1998). This frequency limit is further supported by normative data published within the TFA CARNet White Paper which noted MCA phase values in the White Paper decreased from 0.05 Hz (0.82) to 0.10 Hz (0.74), while gain values increased from 0.05 Hz (0.68) to 0.10 Hz (0.83) (Panerai et al., 2023). Nevertheless, it is important to note that the degree of counter‐regulation may differ between vessels and cardiac cycle components at the same frequency. For example, compared to the normative TFA values in the middle cerebral artery, posterior cerebral artery phase values were greater at both frequencies (0.05 Hz: 0.38 and 0.10 Hz: 0.56) and the gain values were lower (0.05 Hz: 0.38 and 0.10 Hz: 0.56). Further, compared to mean, diastole displayed less regulation with lower phase values of 0.77 at 0.05 and 0.68 at 0.10 Hz and greater gain values of 0.89 at 0.05 and 1.07 at 0.10 Hz. However, consistent with previous work, the greatest regulation occurring within the cerebral pressure‐flow relationship occurs at systole with phase values of 1.23 at 0.05 and 1.02 at 0.10 Hz and gain values of 0.41 at 0.05 and 0.48 at 0.10 Hz. In summary, it appears using a singular value as the upper‐frequency limit of autoregulation is an oversimplification, as this may additionally be influenced by other confounding variables such as sex, age, clinical status, and so forth. Given the complexity of CA, several methods of analysis combining frequency, time, and non‐linear methods are likely required to pinpoint a specific autoregulatory threshold. A limitation of the current investigation is the majority of the sample was comprised of males (89%). Nonetheless, the absence of an autoregulatory plateau in ~70%–80% of datasets would likely be nominally influenced by sex.

In conclusion, poor levels of reliability were consistently noted when attempting to delineate the CA plateau across the cardiac cycle through PPR analysis. This may be due to the notion the methodological approach employed with PPR does not always result in clear and consistent evidence of a central plateau with distinctive rising and falling slopes. Given the lack of reliability found in this study (despite the same data set showing highly reproducible results for transfer function analysis) (Smirl et al., 2015) and the physiological complexities known to exist within the cerebral pressure‐flow relationship (i.e., differences between vessels, frequencies, and cardiac cycle components), these results demonstrate caution should be used when employing this method to make between‐group and within‐group inferences. This minimizes the physiological and clinical utility of PPR for further understanding disease progression in those with known CA impairments.

## FUNDING INFORMATION

J.S.B received funding from the University of Calgary (John D Petrie QC Memorial Scholarship) and the Natural Sciences and Engineering Research Council (CGSD3559333‐2021; Alexander Graham Bell Canada Graduate Scholarship‐Doctoral Program). J.D.S. received funding from the Natural Sciences and Engineering Research Council (RGPIN‐2020‐04057) and Killam Predoctoral Fellowships.

## CONFLICT OF INTEREST STATEMENT

The authors declare they have no conflicts of interest to report.

## Data Availability

Data will be shared upon reasonable request to corresponding author (J.S.B.).
